# Adequate application of recombinant thrombomodulin for sepsis-associated disseminated intravascular coagulation

**DOI:** 10.1186/s13054-015-0944-3

**Published:** 2015-05-22

**Authors:** Toshiaki Iba

**Affiliations:** Department of Emergency and Disaster Medicine, Juntendo University Graduate School of Medicine, 2-1-1 Hongo, Bunkyo-ku, Tokyo, 113-8421 Japan

Recombinant thrombomodulin (rTM) is widely used for septic disseminated intravascular coagulation (DIC) in Japan. Tagami and colleagues performed an analysis using a nationwide administrative database in Japan and reported that rTM did not reduce the mortality in pneumonia-associated DIC (37.6 % in the rTM group vs 37.0 % in the control group, odds ratio: 1.01 (95 % confidence interval: 0.93 to 1.10)) [1]. They also reported a similar result in patients with bowel perforation-induced DIC [2]. These reports indicate that rTM will not be effective in real clinical practice. In contrast, in a recent issue of *Critical Care*, Yoshimura and colleagues reported that administration of rTM was significantly associated with reduced mortality in high-risk patients [3]. The same group also reported in their systematic review that the probability of a beneficial effect with rTM increases with increasing baseline risk [4].

Based on these findings, I suppose that rTM is not properly used currently. The decision based on Japanese Association of Acute Medicine DIC diagnostic criteria may not be adequate. As a matter of fact, Tagami and colleagues performed a very similar analysis for antithrombin concentrate and reported its efficacy (odds ratio: 0.85 (95 % confidence interval: 0.75 to 0.97)) [5]. We must remember that the Japanese Association of Acute Medicine criteria were designed specifically with the purpose of helping physicians decide on the timing of anticoagulant treatment, and antithrombin was the primary anticoagulant at that time. I therefore propose establishing different criteria, such as ‘fulfill the Japanese Association of Acute Medicine DIC criteria and Acute Physiology and Chronic Health Evaluation II score >24’, for the application of rTM.

## Abbreviations

DIC: disseminated intravascular coagulation
rTM: recombinant thrombomodulin

## References

[1] Tagami T, Matsui H, Horiguchi H, Fushimi K, Yasunaga H (2015). Recombinant human soluble thrombomodulin and mortality in severe pneumonia patients with sepsis-associated disseminated intravascular coagulation: an observational nationwide study. J Thromb Haemost.

[2] Tagami T, Matsui H, Fushimi K, Yasunaga H (2015). Use of recombinant human soluble thrombomodulin in patients with sepsis-induced disseminated intravascular coagulation after intestinal perforation. Font Med.

[3] Yoshimura J, Yamakawa K, Ogura H, Umemura Y, Takahashi H, Morikawa M (2015). Benefit profile of recombinant human soluble thrombomodulin in sepsis-induced disseminated intravascular coagulation: a multicenter propensity score analysis. Crit Care.

[4] Yamakawa K, Aihara M, Ogura H, Yuhara H, Hamasaki T, Shimazu T (2015). Recombinant human soluble thrombomodulin in severe sepsis: a systematic review and meta-analysis. J Thromb Haemost.

[5] Tagami T, Matsui H, Horiguchi H, Fushimi K, Yasunaga H (2014). Antithrombin and mortality in severe pneumonia patients with sepsis-associated disseminated intravascular coagulation: an observational nationwide study. J Thromb Haemost.

